# Reading the Mind in the Eyes of Children Test (RME-C-T): Development and Validation of a Complex Emotion Recognition Test

**DOI:** 10.3389/fpsyt.2020.00376

**Published:** 2020-05-20

**Authors:** Rike Pahnke, Anett Mau-Moeller, Alfons O. Hamm, Alexander Lischke

**Affiliations:** ^1^Institute of Sport Science, University of Rostock, Rostock, Germany; ^2^Department of Psychology, University of Greifswald, Greifswald, Germany

**Keywords:** mindreading, emotion recognition, empathy, perspective taking, empathetic concern

## Abstract

Much research has been devoted to the development of emotion recognition tests that can be used to investigate how individuals identify and discriminate emotional expressions of other individuals. One of the most prominent emotion recognition tests is the Reading the Mind in the Eyes Test (RME-T). The original RME-T has been widely used to investigate how individuals recognize complex emotional expressions from the eye region of adult faces. However, the RME-T can only be used to investigate inter-individual differences in complex emotion recognition during the processing of adult faces. To extend its usefulness, we developed a modified version of the RME-T, the Reading the Mind in the Eyes of Children Test (RME-C-T). The RME-C-T can be used to investigate how individuals recognize complex emotional expressions from the eye region of child faces. However, the validity of the RME-C-T has not been evaluated yet. We, thus, administered the RME-C-T together with the RME-T to a sample of healthy adult participants (*n* = 119). The Interpersonal Reactivity Index (IRI) and the Toronto Alexithymia Scale (TAS) were also administered. Participants’ RME-C-T performance correlated with participants’ RME-T performance, implying that the RME-C-T measures similar emotion recognition abilities as the RME-T. Participants’ RME-C-T performance also correlated with participants’ IRI and TAS scores, indicating that these emotion recognition abilities are affected by empathetic and alexithymic traits. Moreover, participants’ RME-C-T performance differed between participants with high and low TAS scores, suggesting that the RME-C-T is sensitive enough to detect impairments in these emotion recognition abilities. The RME-C-T, thus, turned out to be a valid measure of inter-individual differences in complex emotion recognition during the processing of child faces.

## Introduction

Every day we have to interact with other individuals. Sometimes these individuals are unable or unwilling to give us detailed information about their emotional condition. This makes it difficult for us to act and react in an appropriate manner. However, we can try to make inferences about their emotional condition on the basis of their facial expressions. Facial expressions are difficult to control (1, 2), implying that they convey important information about the emotional states of our interaction partners (3). Using this information allows us to adapt our actions and reactions to their emotional needs, which is essential for the establishment of mutual understanding. Those of us who have difficulties to use this information also have difficulties to understand their interaction partners, indicating that the course of our interactions is crucially affected by the way we process information that is provided by the facial expressions of our interaction partners.

Accumulating evidence suggest that this is indeed the case (3). Individuals who have difficulties to use the facial expressions of their interaction partners for emotional state inferences also have difficulties to initiate and maintain interpersonal relationships with these interaction partners. This becomes most obvious in the context of neuropsychiatric, neurodevelopmental, and neurodegenerative disorders that are characterized by interpersonal difficulties (4). Individuals with schizophrenia, autism, or Alzheimer disease, for instance, frequently experience interpersonal conflicts that are due to a misperception or misinterpretation of emotional expressions (5–7). Consequently, much research has been devoted to the development of tests that help to elucidate the relationship between face processing and interpersonal conflicts (8–10).

Although these tests provided important insights into the dynamics of interpersonal conflicts (3), the insights have been limited to interpersonal conflicts that involve adults. Little is known about the dynamics of interpersonal conflicts that involve children because there is a lack of tests that are suited to investigate how individuals process facial expressions of children. However, a misperception or misinterpretation of children’s emotional expression increases the likelihood of interpersonal conflicts in adult-child contexts (11). Abusive individuals, for instance, often engage in punitive behavior because they mistake children’s neutral expressions for hostile ones (12, 13). Considering that deficits in face processing may have such severe consequence on children’s welfare highlights the need to develop tests that help to investigate how individuals process children’s facial expressions.

There are a few tests available that can be used for such investigations (14, 15). Most of these tests are modifications of tests that are used to investigate how individuals process adults’ facial expressions (8–10). These tests require the discrimination or identification of basic expressions that are prototypical exemplars of emotions like anger, disgust, fear, sadness, or happiness (16). Although these basic emotions can be easily discriminated and identified (17), they are rarely experienced and expressed in real life (18). The experience and expression of complex emotions, which are often blends of basic emotions (e.g., shame as mixture of fear and disgust), is far more prevalent (18). Tests requiring the discrimination or identification of complex emotional expressions are, thus, more useful to investigate how individuals process children’s facial expressions. However, these types of tests are currently not available.

In consideration of this, we developed a test that requires the identification of complex emotional expressions in faces of children. Our test is a modification of the well-known Reading the Mind in the Eyes Test (RME-T) (19), a complex emotion recognition test that involves the presentation of adult faces^1^. The RME-T requires the identification of emotional expression on basis of information that is conveyed by the eye region. In order to be identified, the emotional expressions have to be matched with emotional states that describe the emotional expressions in a correct manner. The identification process is quite challenging, making the RME-T far more difficult than tests that require the idenfication of emotional expressions on basis of information that is not limited to the eye region (21, 22). Due to these differences in test difficulty, the RME-T is better suited to investigate how individuals process adult’s facial expressions than other tests. We, thus, thought that it may be worthwhile to develop a modified version of the RME-T for investigations that are concernd with the way individuals process children’s facial expressions.

Our modified version of the RME-T, the Reading the Mind in the Eyes of Children Test [RME-C-T; (23)]^2^, comprises eye regions of child instead of adult faces. Similar as in the RME-T, these eye regions are showing emotional expressions that have to be matched with emotional states that correctly describe the emotional expressions. In order to validate the RME-C-T, we administered the RME-C-T together with the RME-T to a sample of healthy adult participants. Considering that the RME-C-T was developed on basis of the RME-T, we expected participants’ performance on the RME-C-T to correlate positively with participants’ performance on the RME-T. We also administered the Interpersonal Reactivity Index (IRI) (25), a questionnaire measuring empathetic traits like empathetic concern and empathetic perspective-taking. As these empathetic traits facilitate emotion recognition (26, 27), we expected participants’ performance on the RME-C-T to correlate positively with participants’ IRI scores. In addition to the IRI, we administered the Toronto Alexithymia Scale (TAS) (28, 29). This questionnaire measures alexithymic traits like difficulties in identifying or describing one’s own emotions and an externally oriented thinking style. As these alexithymic traits impair emotion recognition (27, 30), we expected participants’ RME-C-T performance to correlate negatively with participants’ TAS scores. Moreover, we expected participants with low TAS scores to perform better on the RME-C-T than participants with high TAS scores. Besides the IRI and TAS, we administered a verbal intelligence test [Mehrfachwortschatztest, MWT-B; (31)] to explore whether participants’ RME-C-T performance correlated positively with participants’ MTW-B scores (32).

## Methods

### Participants

Using public advertisment asking for healthy adults with an interest in psychological studies, we recruited 119 participants (ethnicity: Caucasian; sex distribuition: 86 females, 33 males; age range: 18 to 35 years; educational level: medium to higher education) for the study. We only considered participants for recruitment who were aged between 18 and 35 years, who were native speakers and who passed a screening for neuropsychiatric, neurodevelopmental, and neurodegenerative disorders. A power analysis with G*Power (33) indicated that a sample size of 119 participants provided enough power to detect small to medium sized correlations (*r* = 0.30) between participants’ RME-C-T, RME-T, IRI, TAS, and MWT-B performance and to dectect medium sized differences (*r* = 0.30) in RME-C-T performance between participants with low and high TAS score (1-*β* = 80, *α* = 0.05, one-sided). All participants provided written informed consent to the study protocol that was approved by the ethics commitee of the University of Rostock and the University of Greifswald. The study protocol was carried out in accordance with the Declaration of Helsinki.

### Multiple Vocabulary Test

The Multiple Vocabulary Test (MWT-B) (31) required participants to identify correct words among a series of incorrect words. The number of correctly identified words was determined and used as a measure of participants’ verbal intelligence quotient (MWT-B-IQ). This measure displays good psychometric properties in terms of validity and reliability because it allows a reliable discrimination of individuals with different intelligence levels (31, 34).

### Interpersonal Reactivity Index

The Interpersonal Reactivity Index (IRI) (25) required participants to make statements about other-directed thoughts and feelings (e.g., *When I*’*m upset at someone, I usually try to “put myself in his shoes” for a while*). Participants had to indicate their agreement with each statement on a scale that ranged from 0 (*not true for me*) to 4 (*true for me*). On basis of these statements, measures of participants’ empathetic concern (IRI-EC) and participants’ empathetic perspective-taking (IRI-PT) were determined. These measures can be used for a reliable discrimination of empathetic and non-empathetic individuals (25, 35), indicating good psychometric properties in terms of validity and reliability.

### Toronto Alexityhmia Scale

The Toronto Alexityhmia Scale (TAS) (28, 29) required participants to make statements about self-directed thoughts and feelings (e.g., *I am often confused about what emotion I am feeling*). Participants had to indicate their agreement with each statement on a scale that ranged from 1 (*not true for me*) to 5 (*true for me*). On basis of these statements, measures of participants’ difficulties to identify or describe their own emotions (TAS-DIF, TAS-DDF) and participants’ externally oriented thinking style (TAS-EOT) were determined. These measures possess good psychometric properties in terms of validity and reliability because they allow a reliable discrimination of alexithymic and non-alexithymic individuals (28, 29, 36).

### Reading the Mind in the Eyes Test

The RME-T (19) required participants to recognize complex emotional expressions on basis of information that is provided by the eye region of adult faces. More specifically, participants had to identify emotional expressions by selecting labels that describe distinct emotional states. The eye regions showing the emotional expressions were culled from magazine pictures of young and old adults (17 females, 19 males) and the labels describing the emotional states were compiled by researchers. The labels described either emotional states that matched the expressed emotions (target states) or emotional states that did not match the expressed emotions (distractor states). The match or mismatch between a particular state and a particular expression was determined on a basis of a consensus rating. By pairing each eye region with labels describing states that matched or did not match the expressions, the researchers were able to construct a test version that was sensitive enough to reveal differences in complex emotion recognition during the processing of adult faces (37–41). The test version can be used for a reliable differentiation of individuals with impaired and intact emotion recognition abilities (19, 42, 43), indicating satisfying psychometric properties in terms of validity and reliability.

**Figure 1:**
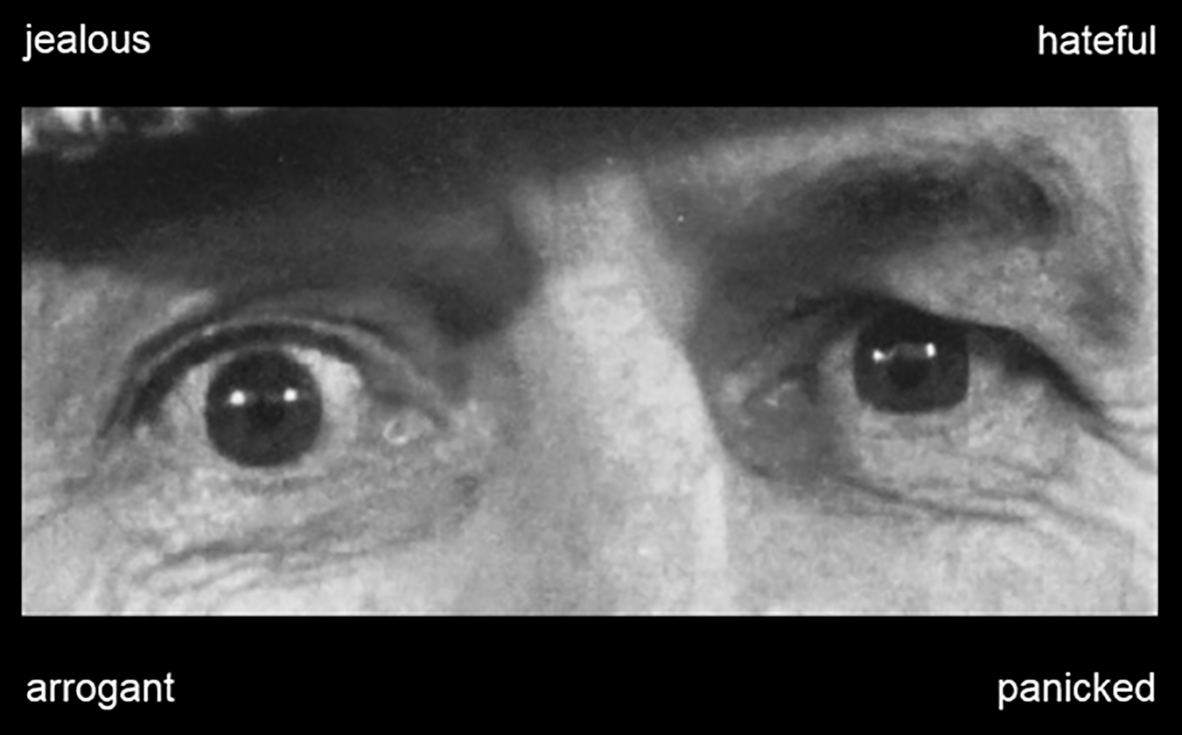
Example of a picture that was used in the original Reading the Mind in the Eyes Test [RME-T, (19)]. The picture shows an eye region expressing a distinct emotional state and labels describing a range of emotional states (one target state, three distractor states). Participants had to identify the label that correctly described the state of the expressed emotion (panicked).

We used a computerized version of the RME-T (41, 44, 45) to present the eye regions and labels to the participants. Thirty-six eye regions showing a range of different emotional expressions were presented in a random order. Each eye region was paired with four labels describing distinct emotional states (one target state, three distractor states). Participants had to select the label that correctly described the state of the expression. No time limit was imposed, but participants were encouraged to select the labels as fast as possible. The number of correctly identified expressions was used as a measure of participants’ ability to recognize complex emotional expressions of adults (**Figure 1**).

### Reading the Mind in the Eyes of Children Test

The Reading the Mind in the Eyes of Children Test (RME-C-T) (23) required participants to recognize complex emotional expressions on basis of information that is provided by the eye region of child faces. Similarly as in the RME-T (19), the emotional expressions had to be identified *via* a selection of labels that describe distinct emotional states. However, the eye regions showing the emotional expressions were not culled from magazine pictures of adults. The eye regions were selected from a pool of pictures of children who had been extensively trained to express the emotional states of the RME-T (19). The picture pool was generated by inviting 30 children (ethnicity: Caucasian; sex distribution: 15 males, 15 females; age range: 8–10 years; educational level: 4th grade) of an elementary school to a photo session that was run by a researcher with expertise in teaching children (RP). The children received information about emotional states and emotional expressions before they were trained to express distinct emotional states. Following established procedures (Ebner, Riediger, & Lindenberger, 2010), the researcher used several techniques to train children’s emotion expression abilities: They heard stories describing an event that caused a particular emotional state, they were asked to describe a personal event that caused the emotional state, and they were asked to re-experience the emotional state by reliving the personal event in their imagination. While re-experiencing the emotional state, they received verbal and non-verbal instructions regarding its expression. Using a high-quality digital photocamera (Canon EOS 20D, Canon, Tokio, Japan), more than 5,000 pictures were taken during a professional photo session that was run by a researcher with expertise in photography (RP). After the photo session, two independent researchers (AL, RP) selected pictures of children who were able to express the prescribed states in an unambigious manner. Following a consensus rating, 676 pictures were deemed suiteable for further evaluation. Before the pictures were subjected to an evaluation study, they were edited with Adobe Photoshop CS4 (Adobe Systems Inc., San Jose, CA, USA) by a researcher with expertise in photography (RP). Following color and contrast correction, the pictures were cropped in a way that only the eye region of the expression remained visible (692 × 346 pixel). One hundred and one healthy adult (ethnicity: Caucasian; sex distribution: 48 females, 53 males; age rage: 18 to 35 years; educational level: higher education) participated in the evaluation study. An in-house interview was used to rule out that the participants suffered from neuropsychiatric, neurodevelopmental, or neurodegenerative disorders (44). All participants were native speakers. For each picture, participants had to indicate whether the depicted expression matched the prescribed state on scale that ranged from 0 (*imperfect match*) to 7 (*perfect match*). There was a high agreement between participants’ ratings (range of intra-class correlation coefficients: 0.922–0.932). Of the 676 pictures, 351 pictures received a minimum score of 5. These pictures were selected for the test constuction. The prescribed states that were associated with the depicted expressions constituted the target states. The target states were paired with distractor states that were compiled by two independent researchers (AL, RP). The target and distractor states were selected from a pool of states that were used in the RME-T (19). By combining the target and distractor states with the expressions of the selected pictures, the researchers tried to develop a test version that comprised an equal number of pictures showing male and female children that expressed the target states with comparable clarity. Following a consensus rating, 34 pictures showing 17 male and 17 female children that expressed the target states in an unambigous manner (rating range of prescribed target states: 5.03–6.78) were determined. This test version appeared suiteable to investigate differences in complex emotion recognition during the processing of child faces. For this reason, it was selected for the present validation study.

**Figure 2:**
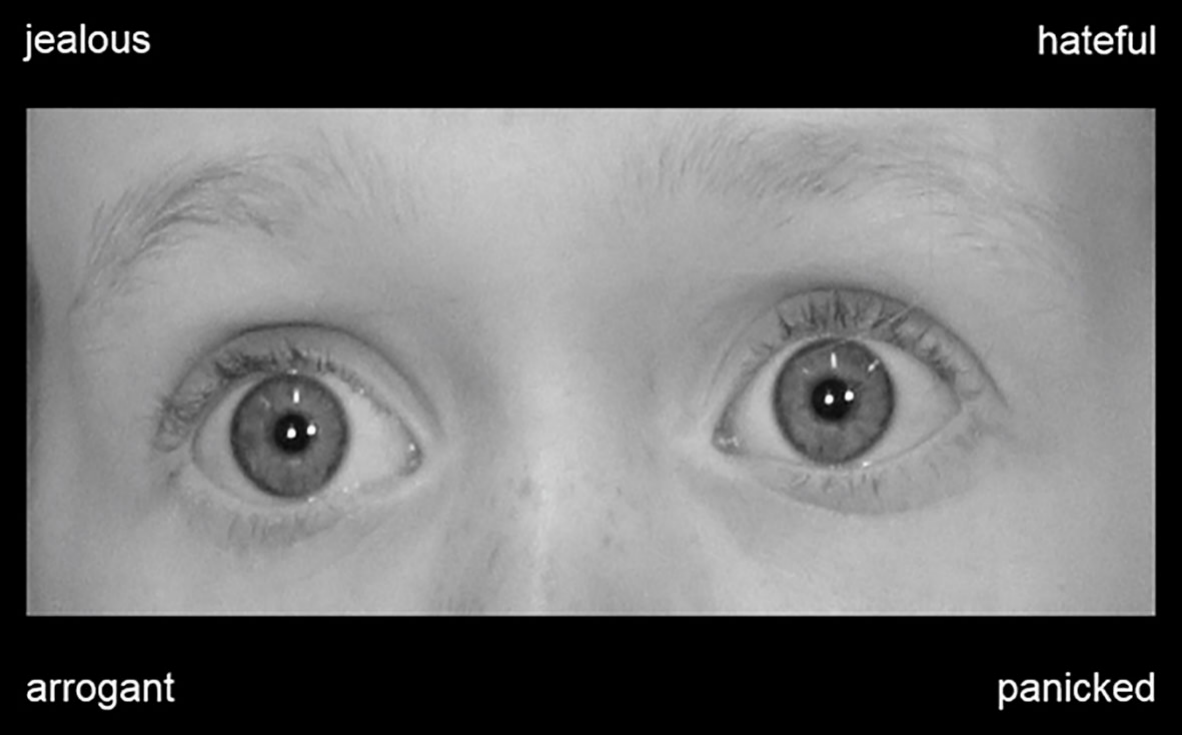
Example of a picture that was used in the Reading the Mind in the Eyes of Children Test [RME-C-T, (23)]. The picture shows an eye region expressing a distinct emotional state and labels describing a range of emotional states (one target state, three distractor states). Participants had to identify the label that correctly described the state of the expressed emotion (panicked).

We used a computerized version of the RME-C-T (23) to present the eye regions and labels to the participants. Thirty-four eye regions showing a range of different emotional expressions were presented in a random order. Each eye region was presented together with four labels describing different emotional states (1 target state, 3 distractor states). Similar as in the RME-T (19), participants had to select the label that correctly described the state of the expression as fast as possible. The number of correctly identified expressions was used as a measure of participants’ ability to recognize complex expressions of children (**Figure 2**).

### Statistical Analysis

Participants’ data were analyzed with SPSS 22 (IBM Corp., Armonk, NY, USA) and R (R Development Core Team, 2018). For preliminary analyses, we ran Wilcoxon tests with Monte Carlo simulations (10,000 samples) to investigate differences in participants’ RME-C-T and RME-T performance. For exploratory analyses, we ran Mann-Whitney tests with Monte Carlo Simulations (10,000 samples) to investigate sex-differences in participants’ RME-C-T, RME-T, IRI, TAS, and MWT-B performance. There were, however, no substantial evidence for such sex differences. We, thus, refrained from further investigating sex-differences in our hypothesis-driven analyses. For these analyses, we ran Spearman correlations to investigate correlations between participants’ RME-C-T, RME-T, IRI, TAS, and MWT-B-IQ performance. In addition, we ran Mann-Whitney Tests with Monte Carlo Simulations (10,000 samples) to investate differences in RME-C-T performance between participants with low and high TAS scores using established cutoff values [non-alexithymic participants: TAS ≤ 51, alexithymic individuals TAS > 51; (46)]. The significance level for all analyses was set at α ≤ 0.05, two-sided for exploratory, and one-sided for hypothesis-driven analyses. Significance values (*p*), effect size measures (*r*), and 95% confidence intervalls (95% CIs) (bootstrapping 10,000 samples) were determined to facilitate the interpretation of these analyses (47).

## Results

### Preliminary Analyses of RME-C-T and RME-T Performance

Participants performed well on the RME-C-T and the RME-T (see **Table 1**), indicating that the RME-C-T and RME-T had a moderate test difficulty. Nontheless, there were differences in participants’ RME-C-T and RME-T performance (*z* = −2.27, *p* = 0.024, *r* = −0.21, 95% CI [-.38, -.03]; see **Table 1**). Participants were better in recognizing complex emotional expressions in child faces than in adult faces. Of note, participants’ performance on the RME-C-T was generally good, regardless whether they processed complex emotional expressions in male or female child faces (*z* = −0.42, *p* = 0.660, *r* = −0.04, 95% CI [-.21, .14]).

**Table 1:** Participant characteristics.

	All participants (*N* = 119)		Female participants (*n* = 86)		Male participants (*n* = 33)	
	*M (SEM)*	95% CI	*M (SEM)*	95% CI	*M (SEM)*	95% CI
Age	21.49 (0.41)	[20.73, 22.37]	21.25 (0.42)	[20.46, 22.03]	22.56 (1.23)	[20.25, 25.00]
MTW-B-IQ^a^	98.43 (0.83)	[96.88, 100.12]	97.83 (0.75)	[96.49, 99.27]	101.11 (3.04)	[95.60, 1 07.00]
IRI-PT	18.55 (0.39)	[17.77, 19.31]	18.90 (0.42)	[18.05, 19.74]	17.00 (0.90)	[15.33, 18.75]
IRI-EC	17.16 (0.54)	[16.09, 18.15]	17.30 (0.59)	[16.17, 18.45]	16.56 (1.44)	[13.63, 19.43]
TAS-DIF	12.27 (0.60)	[11.13, 13.57]	12.10 (0.67)	[10.81, 13.44]	13.00 (1.40)	[10.43, 15.73]
TAS-DDF	10.33 (0.60)	[9.23, 11.55]	10.28 (0.70)	[9.05, 11.74]	10.56 (1.12)	[8.50, 12.64]
TAS-EOT	14.47 (0.45)	[13.57, 15.39]	14.25 (0.48)	[13.30, 15.19]	15.44 (1.21)	[13.22, 17.80]
RME-T	0.71 (0.01)	[0.68, 0.73]	0.71 (0.01)	[0.68, 0.73]	0.71 (0.03)	[0.66, 0.76]
RME-C-T	0.73 (0.01)	[0.71, 0.76]	0.73 (0.01)	[0.71, 0.75]	0.74 (0.04)	[0.64, 0.80]

MWT-B, Multiple Vocabulary Intelligence Test-Verbal Intelligence Quotient (31); IRI-PT, Interpersonal Reactivity Index-Perspective Taking (25); IRI-EC, Interpersonal Reactivity Index-Empathetic Concern (25); TAS-DIF, Toronto Alexithymia Scale-Difficulties Identifying Feelings (28, 29); TAS-DDF, Toronto Alexithymia Scale-Difficulties Describing Feelings (28, 29); Toronto Alexithymia Scale-Externally Oriented Thinking Style (28, 29); RME-T, Reading the Mind in the Eyes Test (19); RME-C-T, Reading the Mind in the Eyes of Children Test (23).

a Data were only available for 49 participants.

### Exploratory Analyses of Sex-Dependent Differences in RME-C-T, RME-T, IRI, and TAS Performance

We found no sex differences in participants’ performance on the RME-C-T (*z* = −1.21, *p* = 0.239, *r* = −0.11, 95% CI [-.28, .07]; see **Table 1**) or RME-T (*z* = −0.18, *p* = 0.849, *r* = −.02, 95% CI [-.20, .16]; see **Table 1**), indicating that male and female participants’ did not differ in their ability to recognize complex emotional expressions in adult or child faces. There were, however, sex differences in participants’ IRI scores. Whereas the ability to adopt others’ perspective was similarly pronounced in male and female participants (IRI-PT: *z* = −0.24, *p* = 0.809, *r* = −0.02, 95% CI [-.19, .16]; see **Table 1**), the ability to feel compassion for others’ emotions was more pronounced in female as compared to male participants (IRI-EC: *z* = −2.83, *p* = 0.008, *r* = −0.26, 95% CI [-.42, -.08]; see **Table 1**). There were also sex-differences in participants’ TAS scores. Whereas male and female participants had comparable difficulties in describing or identifying their own emotions (TAS-DIF: *z* = −0.64, *p* = 0.518, *r* = −0.06, 95% CI [-.24, .12]; TAS-DDF: *z* = −1.51, *p* = 0.110, *r* = −0.14, 95% CI [-.31, .04]; see **Table 1**), male participants showed a more externally oriented thinking style than female participants (TAS-EOT: *z* = −2.97, *p* = 0.003, *r* = −0.27, 95% CI [-.42, -.10]; see **Table 1**).

### Hypothesis-Driven Analyses of Correlations Between RME-C-T and RME-T Performance

**Figure 3:**
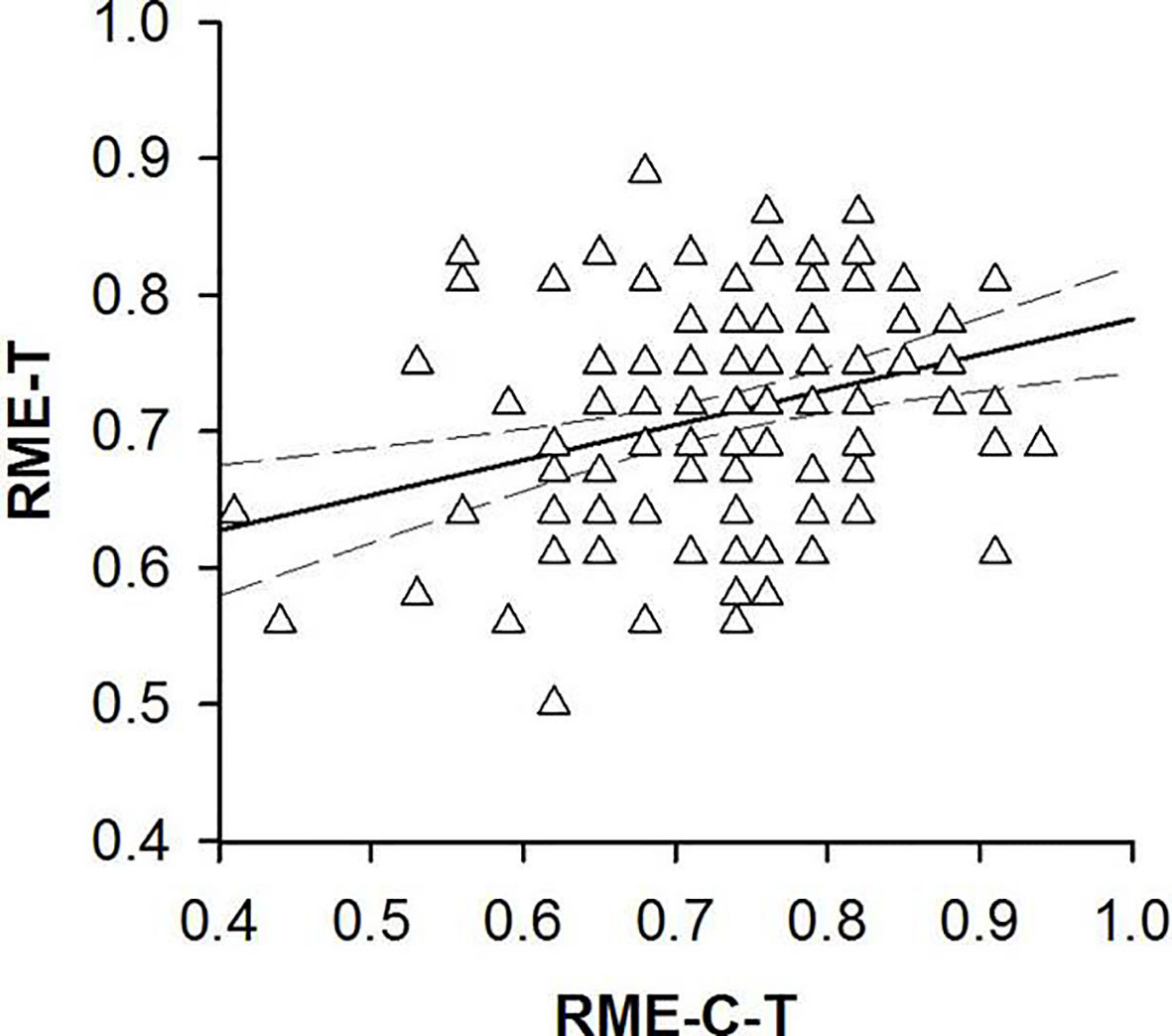
Scatterplots with lines of best fit demonstrating correlations between participants’ performance on the Reading the Mind in the Eyes of Children Test [RME-C-T, (23)] and participants’ performance on the Reading the Mind in the Eyes Test [RME-T, (19)].

We found correlations between participants’ performance on the RME-C-T and RMET. Participants’ ability to recognize complex emotional expressions in child faces correlated positively with participants’ ability to recognize complex emotional expressions in adult faces (*r* = 0.27, *p* = 0.002, 95% CI [.08, .44]; see **Figure 3**).

### Hypothesis-Driven Analyses of Correlations Between RME-C-T, IRI, TAS, and MWT-B Performance

**Figure 4:**
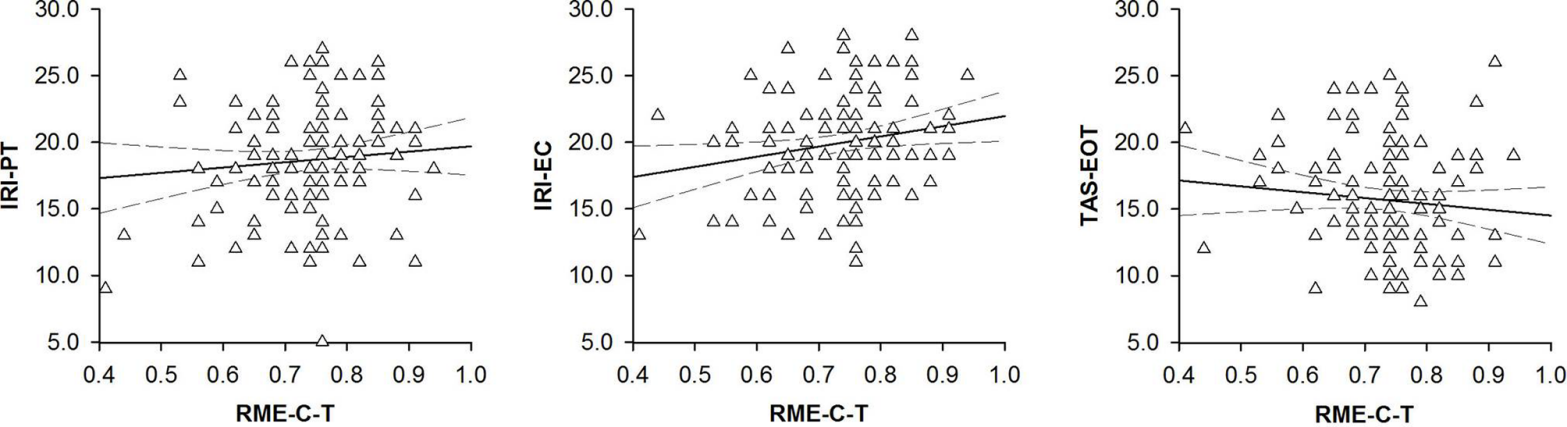
Scatterplots with lines of best fit demonstrating correlations between participants’ performance on the Reading the Mind in the Eyes of Children Test [RME-C-T, (23)] and (*left panel*) participants’ scores on the Interpersonal Reactivity Index-Perspective Taking Scale [IRI-PT, (25)], (*middle panel*), participants’ scores on the Interpersonal Reactivity Index-Empathetic Concern Scale [IRI-EC, (25)] and (*right panel*) participants’ scores on the Toronto Alexithymia Scale-Externally Oriented Thinking [TAS-EOT, (28, 29)].

We found correlations between participants’ RME-C-T performance and participants’ IRI scores. Participants ability to recognize complex expressions in child faces correlated positively with participants’ ability to adopt others’ perspective (IRI-PT: *r* = 0.17, *p* = 0.033, 95% CI [.00, .36]; see **Figure 4**) as well as with participants’ ability to feel compassion for others’ emotions (IRI-EC: *r* = 0.19, *p* = 0.018, 95% CI [.02, .36]; see **Figure 4**). We also found correlations between participants’ RME-C-T performance and participants’ TAS scores. Participants’ ability to recognize complex emotional expressions in child faces correlated negatively with participants’ externally oriented thinking style (TAS-EOT: *r* = −0.22, *p* = 0.002, 95% CI [-.39, -.03]; see **Figure 4**) but not with participants’ difficulties in identifying or describing their own emotions (TAS-DIF: *r* = −0.10, *p* = 0.297, 95% CI [-.28, .08]; TAS-DDF: *r* = 0.00, *p* = 0.961, 95% CI[-.18, .19]). In contrast, we found no correlations between participants’ RME-C-T performance and participants’ MTW-B scores. Participants’ ability to recognize complex emotional expressions in child faces was uncorrelated with participants’ verbal intelligence quotient (MWT-B-IQ: *r* = 0.10, *p* = 0.486, 95% CI [-.22, .38]).

### Hypothesis-Driven Analyses of Alexithymia-Dependent Differences in RME-C-T Performance

**Figure 5:**
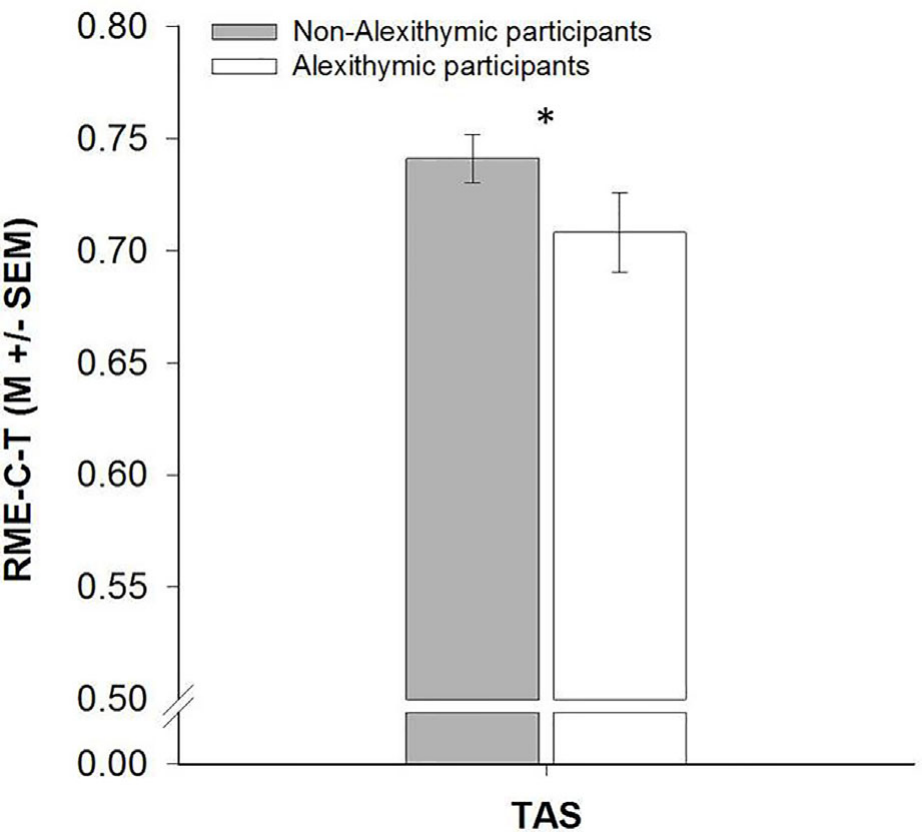
Barplots demonstrating differences in Reading the Mind in the Eyes of Children [RME-C-T, (23)] performance between participants with high and low scores on the Toronto Alexithymia Scale [TAS, (28, 29)].

We found that participants with low TAS scores perfomed better on the RME-C-T than participants with high TAS scores (TAS: *z* = −2.24, *p* = 0.009, *r* = −0.22, 95% CI [-.38, -.04]; see **Figure 5**). Non-alexithymic participants were better in recognizing complex emotional expressions in child faces than alexithymic individuals.

## Discussion

The RME-T (19) has been extensively used to investigate how individuals process complex emotional expressions on basis of information that is provided by the eye region of adult faces (37–41, 45). To extend its usefulness, we developed a modified version of the RME-T, the RME-C-T (23), that can be used to investigate how individuals process complex emotional expressions on basis of information that is provided by the eye region of child faces. Although the development of the RME-C-T was based on the same procedure as the development of the RME-T, there were notable differences in the development of the RME-C-T and RME-T. For the RME-T, the eye regions were taken from magazine pictures of young and old adults (19). These black and white pictures differed markedly in image quality, making it difficult to recognize the emotional states that were expressed by the eye regions (48). The emotional state conveyed by each expression was determined on basis of a consensus rating (19), leaving open whether the state that was assigned to an expression actually corresponded with the state that caused this expression (49). For the RME-C-T, the eye regions were taken from pictures of young children who were extensively trained to express the emotional states. These color pictures were carefully edited to rule out that differences in image quality affected the recognition of the emotional states that were expressed by the eye regions. The emotional state conveyed by each expression was determined on basis of consensus and empirical ratings, implying a correspondence between the state that was assigned to an expression and the state that caused this expression. Taking these differences in test development into account, we assumed that there was less ambiguity regarding the expression of the emotional states in the RME-C-T than in the RME-T. Our analyses regarding differences between participants’ RME-C-T and RME-T performance supported these assumptions. Participants were more accurate in the recognition of complex emotional expressions in the RME-C-T than in the RME-T. The respective analyses, thus, provided initial evidence for the validity of the RME-C-T (50).

We found further evidence for the validity of the RME-C-T in our analyses regarding correlations between participants’ RME-C-T and RME-T performance (50). Participants’ performance on the RME-C-T correlated positively with participants’ performance on the RME-T. The positive nature of the correlation implies that the RME-C-T and RME-T measure similar emotion recognition abilities, namely abilities that are necessary for the recognition of complex emotional expressions on basis of information that is provided by the eye region of faces. The size of the correlation fell in the small to medium range (47), indicating that the RME-C-T and RME-T measure these emotion recognition abilities in different ways. These measurement differences are probably due to the aforementioned differences in test development (i.e., use of low-quality black and white pictures of faces showing young and old adults with ambiguous expressions, use of high-quality color pictures of faces showing young children with unambiguous expressions). However, correlations among different emotion recognition tests usually turn out to be of small size because of measurement-specific differences between these tests (51). The size of the correlation between participants’ RME-C-T and RME-T performance, thus, fell in the expected range of possible sizes.

Our analyses regarding correlations between participants’ performance on the RME-C-T and participants’ scores on the IRI and TAS provided additional evidence for the validity of the RME-C-T (50). Participants’ performance on RME-C-T correlated positively with participants’ IRI scores. The positive nature of the correlation implies that the RME-C-T measures emotion recognition abilities that are affected by empathetic traits that are known to facilitate emotion recognition (26, 27). Participants’ performance on the RME-C-T correlated negatively with participants’ TAS scores. The negative nature of the correlation indicates that the RME-C-T measures emotion recognition abilities that are affected by alexithymic traits that are known to impair emotion recognition (27, 30). The size of these correlations fell in the small range (47), which probably reflects differences in the respective tests (i.e., use of questionnaires for the measurement of empathetic and alexithymic traits, use of performance test for the measurement of emotion recognition abilities). However, correlations among questionnaire-based and performance-based tests of traits or abilities that are relevant for emotion recognition are usually of small size due to measurement-specific differences between these tests (52). The size of the correlations between participants’ RME-C-T performance and IRI or TAS scores, thus, also fell in the expected range of possible sizes.

We found further evidence for the validity of the RME-C-T in our analyses regarding differences in RME-C-T performance between participants with low and high TAS scores (50). Participants with low TAS scores performed better on the RME-C-T than participants with high TAS scores, indicating that the RME-C-T discriminates between non-alexithymic individuals that are known to have intact emotion recognition abilities and alexithymic individuals that are known to have impaired emotion recognition abilities (27, 30). The size of these differences fell in the small to medium range (47), which is not surprising considering that participants were healthy adults who rarely display clinically relevant levels of alexithymia that lead to gross impairments in emotion recognition (53). The size of differences in RME-C-T performance between participants with low and high TAS scores, thus, also fell in the expected range of possible sizes. Moreover, the size of these differences indicates that the RME-C-T is sensitive enough to detect subtle impairments in complex emotion recognition.

Taken together, our findings provide evidence for the validity of the RME-C-T. We found the hypothesized correlations between participants’ RME-C-T and RME-T performance, and we found the hypothesized correlations between participants RME-C-T performance and participants’ IRI or TAS scores, indicating that the RME-C-T measures complex emotion recognition abilities that are affected by empathetic and alexithmic traits (26, 27, 30). We also found the hypothesized differences in RME-C-T performance between participants with low and high TAS scores, indicating that the RME-C-T detects differences in complex emotion recognition abilities that vary as a function of alexithymic traits (27, 30). The size of the aforementioned correlations and differences fell in the range of sizes that could be expected on basis of previous findings in healthy individuals (51, 52, 54). These individuals are more empathetic and less alexithymic than individuals with neurodevelopmental, neuropsychiatric, or neurodegenerative disorders (4). We, thus, assume we would have found larger correlations and differences if we had included individuals with neurodevelopmental, neuropsychiatric, or neurodegenerative disorders in the respective analyses. We will test these assumptions in future investigations that are not restricted to healthy individuals. These types of investigations may also help to characterize the RME-C-T with respect to other psychometric properties than those that are related to validity. Although reliability appeared to be satisfactory (ω = 0.68), we will further investigate the psychometric properties of the RME-C-T. If these investigations turn out to be favorable, we will make the RME-C-T available for other researchers.

We believe that the RME-C-T may be useful for other researchers because there is a lack of emotion recognition tests that can be used to investigate how individuals perceive and interpret emotional expressions in child faces. We see the usefulness of the RME-C-T foremost in research that is concerned with the way adults process emotional expressions of children. However, the RME-C-T may also be useful for researchers who are interested to investigate how children process emotional expressions of other children. The RME-C-T may, thus, be useful for researchers in various fields of psychology, including but not limited to social psychology, clinical psychology, and developmental psychology.

## Data Availability Statement

The datasets generated for this study are available on request to the corresponding authors.

## Ethics Statement

The studies involving human participants were reviewed and approved by the ethics committee of the University of Rostock and the University of Greifswald. The patients/participants provided their written informed consent to participate in this study.

## Author Contributions

AL and RP designed the study, collected the data, analyzed the data, and wrote the manuscript. AH and AM-M contributed to writing, reviewing, and editing of the manuscript. All authors approved the final version of the manuscript.

## Funding

Funding for this study was supported by an Open Access Publishing grant that was provided by the German Research Foundation (DFG) and the University of Rostock. AL was supported by a fund from the German Research Foundation (DFG; LI 2517/2-1). The funding source had no further role in the study design, in the collection, in the analysis, and in the interpretation of the data; in the writing of the manuscript; and in the decision to submit the manuscript for publication.

## Conflict of Interest

The authors declare that the research was conducted in the absence of any commercial or financial relationships that could be construed as a potential conflict of interest.

## Footnotes

1 It should be noted that the RME-T was originally intended to be a theory of mind test (19). However, accumulating evidence suggest that the RME-T is in fact a complex emotion recognition test (20).

2 The RME-C-T should not be confused with the RME-T children’s version (24). The RME-T children’s version is as simplified version of the RME-T that also involves the presentation of adult faces. The RME-C-T (23), on the contrary, involves the presentation of child faces.
